# Educating Medical Students on Deaf-Hearing Interpreter Teams: A Virtual Patient Panel Experience

**DOI:** 10.7759/cureus.85290

**Published:** 2025-06-03

**Authors:** Benedicta O Olonilua, Natalie Snyder, Julia Croce, Dimitrios Papanagnou

**Affiliations:** 1 Department of Medical Education, Thomas Jefferson University, Philadelphia, USA; 2 Department of Otolaryngology, Thomas Jefferson University, Philadelphia, USA; 3 Department of Emergency Medicine, Thomas Jefferson University, Philadelphia, USA

**Keywords:** communication training, cultural competency, patient panel, undergraduate medical education, virtual training

## Abstract

Communication and cultural differences render d/Deaf patients vulnerable to poorer health outcomes when compared to their hearing peers. Interventions designed to address these inequities are a growing focus in medical education, with virtual platforms (e.g., Zoom) becoming increasingly popular. This article describes the implementation of a one-hour virtual patient panel with an interactive question-and-answer session between a certified Deaf interpreter (CDI) and second-year pre-clerkship medical students at the authors’ institution. Following this discussion, students were encouraged to share one or two key takeaways from the session through a survey link. These results were analyzed using generative artificial intelligence to summarize key themes. There were 41 respondents, with a response rate of 15%. The key themes that emerged are grouped under the following thematic headings: role of interpreters and communication; insights about d/Deaf culture; challenges and experiences; and suggestions for improvement. Students’ takeaways emphasized themes of communication, cultural competency, and access to care. Student reflections highlighted the novelty and clinical applicability of lessons learned from the panel for their future practice. Further work in medical education is needed to adequately expose healthcare trainees to Deaf culture, particularly how to work with interpreters and CDIs in the clinical environment.

## Introduction

An estimated 70 million people worldwide are deaf and use sign language to communicate [1]. In the United States alone, approximately 500,000 Americans use American Sign Language (ASL) as their primary communication modality [2]. We differentiate deafness with a lowercase ‘d’ to denote the inability to hear from Deafness with an uppercase ‘D’ to represent a unique linguistic, cultural, and social identity [1]. Communication and cultural differences render d/Deaf patients vulnerable to poorer healthcare outcomes compared to their hearing peers (e.g., poorer health literacy, poorer physical health outcomes, and mental health outcomes) [1,3-5]. Furthermore, d/Deaf patients are more likely to have trust in the healthcare system if they feel that there is recognition and awareness of deaf culture, as well as being provided with services that are culturally competent [6]. Interventions designed to address these inequities are a growing focus in medical education [3,7]. These interventions target various topics, including use of ASL in clinical settings and familiarizing healthcare providers with aspects of Deaf culture to improve communication [3,5,7,8]. In the years following the COVID-19 pandemic, virtual platforms (e.g., Zoom) have been an increasingly popular tool in medical education for disseminating these ideas [7,9]. In this article, we will discuss the importance of cultural competency in medical education and current gaps for Deaf populations, introduce the concept of certified Deaf interpreters (CDIs) and Deaf-interpreter hearing-interpreter teams, and describe the implementation of a virtual patient panel on these topics for pre-clinical medical students at our institution.

Cultural competency is a cornerstone of medical education [10], though according to the National Association of the Deaf, most medical training programs do not adequately prepare medical professionals to communicate effectively with Deaf patients [11]. Despite recommendations in the United States from the Liaison Committee on Medical Education (LCME) to incorporate cultural competency in medical curricula, the needs of Deaf individuals are not highlighted specifically, with one study finding that only 6% of interventions in this realm addressed disability and/or Deaf culture [1,12,13]. Interventions in this realm are crucial, as current literature suggests that Deaf cultural competency training for medical trainees and professionals may improve these patients’ health access, literacy, and outcomes [1,13]. Several interventions to familiarize medical students with this cultural competency and related communication services have been recommended for undergraduate medical education; these include incorporating Deaf patients and interpreters into problem-based learning sessions, Deaf standardized patients for clinical encounters, and lectures that allow students to interface with interpreters [12,13].

A unique aspect of Deaf-hearing cultural competency is Deaf interpreter-hearing interpreter teams, which are commonly used in hospitals and healthcare settings for effective conversations with patients and families. These teams consist of CDIs, who are native ASL users and experts in Deaf culture, and hearing interpreters, who can vocalize and receive spoken information. An example of this process in a healthcare setting is as follows: a hearing clinician begins the conversation by speaking, and their speech is interpreted into ASL for the Deaf interpreter by the hearing interpreter. The Deaf interpreter, in turn, emends this interpretation to make it culturally appropriate so that the Deaf patient can understand. When the Deaf patient responds, their signing is interpreted by the Deaf interpreter who can reformulate the message for the hearing interpreter, who then vocalizes it to the hearing clinician [14]. This type of collaboration optimizes language and culture, enabling the hearing healthcare provider and the Deaf patient to understand one another through mediators (i.e., the hearing and the Deaf interpreter) who are experts in their respective cultures [14,15]. This model is used in a variety of environments, including legal contexts, public news broadcasts, and emergencies, where communication might be particularly challenging [7,14,15]. Current research in this area seems to largely focus on the traditional interpreter model (i.e., a hearing interpreter who is fluent in ASL), with few studies examining the interplay of the CDI in medical contexts [14,15]. Unlike the traditional model where a single hearing interpreter translates spoken language directly into ASL, the CDI team bridges cultural and linguistic gaps, particularly when the Deaf individual may use non-standard sign language, may have limited ASL fluency, or may be in a high-stress and/or sensitive setting. Training in undergraduate medical education is essential to prepare medical students for these nuances in patient communication.

## Technical report

In this technical report, we share our experiences implementing a virtual patient panel in our medical school curriculum that was focused on communicating with Deaf patients. This panel builds on a previous workshop by Snyder et al. that was piloted in 2023 [7]. The didactic portion and learning objectives of the panel remained the same as in the 2023 pilot and aimed to amplify goals aligned with the Health Systems Science thread of the medical school curriculum [7]. For this iteration, our session was presented to 271 second-year medical students during the neurology/psychiatry organ-system block (November of 2024).

The session opened with a brief 5-minute introductory presentation by course faculty. The presentation included a summary of the Americans with Disabilities Act and its relevance to the care of Deaf individuals and their families. Terms were also defined; specifically, the difference between Big 'D' and little 'd' was made. Big ‘D’ Deaf refers to the cultural identity of deaf individuals, while little 'd' deaf refers to the state of hearing loss. The presentation outlined the distinct roles of interpreters in healthcare. The presentation served as a segue for the panel. In its 2024 iteration, the panel session featured one Deaf panelist with decades of experience working as a CDI. The panelist shared her experiences as both a Deaf patient and as a CDI.

The panel highlighted the importance of CDIs, who help patients and their families of the Deaf community navigate both language and cultural nuances. Panelists discussed the concept of Deaf-hearing interpreter teams and emphasized the partnership between CDIs and hearing interpreters that is required to facilitate clear and culturally sensitive communication. Similar to our previous workshop in 2023, students in attendance had the opportunity to participate in a question-and-answer session with the panelists [7].

Once the panel was over, students were encouraged to share several of their takeaways from the session. Specifically, they were asked to reply to a single prompt: "Please share 1-2 take-home points from today's session." This qualitative information was collected through an institutionally-secure, electronic survey link. These results were anonymized and analyzed using generative artificial intelligence to help the team identify major key themes. The team's institutional Microsoft Copilot service was used for this purpose. Members of the study team reviewed the output for accuracy while referencing original data [16]. It is important to note that while this was not a formal qualitative research study, findings from this analysis were purely used for hypothesis generation for subsequent studies and interventions.

Overall, 41 students responded (response rate 15%) to this aforementioned prompt, which limits the generalizability of our findings. As such, we acknowledge that the themes identified may not fully represent the perspectives of our broader student cohort.

## Discussion

Key themes that emerged from students' comments were grouped under the following thematic headings: role of interpreters and communication; insights about d/Deaf culture; challenges and experiences; and suggestions for improvement. These comments were helpful to better understand the impact of the educational panel. We discuss each of these below.

Role of interpreters and communication

Similar to previous studies [14,15], responses highlighted the crucial role of interpreters, specifically the distinction between CDI roles and ASL interpreters, with one student stating that “the role of the CDI is different from an ASL interpreter, and it is incredibly important to have both present to ensure communication between patient and provider.” Participants noted the importance of effective communication in healthcare settings for Deaf patients, pointing out the unique challenges faced by health professionals when interacting with this population; this builds on findings reported in previous studies [1,2,4]. Comments such as “it will be really important to keep these examples in mind as we enter rotations and meet with patients with diverse needs and circumstances” and “hearing the initial story that [the CDI] shared that made her want to get involved…really made me think how important being comfortable with the[se] services is as a physician,” highlight the importance of this type of intervention early in medical students’ training to enhance interactions with patients on clinical clerkships and beyond.

This feedback also emphasized how interpreters help bridge gaps in communication. Bridging this gap is critical as it enables better understanding and care for Deaf individuals [1,5,7,8]. Students highlighted the importance of this panel and similar interventions in medical education, with statements such as “more work needs to be done to educate health care professionals on working with the d/Deaf community” and “I think the curriculum should include more sessions about how to communicate with patients with communication barriers.”

Insights about d/Deaf culture

Similar to other similar sessions described in the literature [5,7,8,12], participants found our session particularly insightful, with many mentioning the value of hearing real-life stories, such as those shared by the panelist, which provided new perspectives. Feedback such as “[I] had no idea some things even existed, like what CDIs [are]” and “this was a fantastic way to start learning more about [the d/Deaf community]” underscore the novelty of this material in students’ education. This information was not only novel, but also “very informative. It taught me essential information that I should know regarding caring for a patient who may be deaf or hard of hearing.”

Other participants appreciated the chance to learn from different points of view, especially on how various healthcare systems approach care for Deaf patients. Many expressed that the session provided a deeper understanding of the complexities involved in supporting Deaf individuals in medical settings, fostering greater empathy and awareness of the roles that deaf-hearing teams play in patient communication [14,15]. This is embodied in one student’s reflection, where they state “I learned a lot about the role of body language in interpreting, about how having [an] IV attached or certain muscle/nerve disorders can affect signing/communication, and the nuances between English and ASL as well as between ASL and other signed language/communication.”

Challenges and experiences

On further review of the feedback provided by students, several commented on their own personal experiences where communication barriers with Deaf patients were evident. One student shared their own challenges: “I ran into a situation where I had learned some ASL and was working with [deaf] Ukrainian refugees and realized I couldn’t understand [their] sign language, which made me realize the variation in sign language[s].” Another student reflected on difficulties navigating language differences in medical practice, stating that “the pandemic and use of masks made sign language interpretation more difficult. Motor impairment, arthritis, loss of digits, and other conditions affecting the hands/fingers can make communicating more challenging for a patient, but that’s why we have CDIs to bridge that gap.” These experiences underscore the need for continued training and awareness around communicating with Deaf patients, which have been extensively described in previous studies [8,12,13]. Many participants also noted the importance of recognizing the unique needs of Deaf patients to ensure they receive appropriate care, with one student noting that “I learned why a CDI is a vital part of being able to communicate effectively with deaf patients.”

Suggestions for improvement

While many students appreciated the session, some suggestions for improvement were offered. Suggestions focused on logistics of the session, such as “consider extending this session to 1.5 hours” or “I think this is a great session to keep online. It was very useful to see the interpreter and [the panelist] at the same time.” This feedback reflects considerations from this panel’s previous iteration, including time management and optimal delivery for students given the time of year of the panel (i.e., close to the Thanksgiving holiday break) [7].

In addition to these logistics, other respondents recommended enhancing the focus on the role of interpreters in clinical settings to ensure better integration of sign language support [14,15]. Some responses, such as “please emphasize a bit more who we should be looking at during an interaction with a deaf person (i.e., them vs. the CDI vs. the hearing interpreter),” highlight students’ desire for increased education in this area and potential opportunities for future intervention (e.g., utilization of Deaf standardized patients or clinical simulation). Similarly, participants suggested more practical training on the logistics and tools available for improving communication in medical contexts with Deaf patients, with one student stating that “it would be great to include more sessions about how to communicate with patients with communication barriers…as these are patients we will see regularly in clinical settings, and I do not feel prepared to give competent care.”

## Conclusions

Our patient panel continues to be well-received by medical students at our institution. Students appreciate facilitated conversations in the curriculum that focus on cultural competency, patient communication, and access to care. Students find these conversations to be directly relevant to the work they will be expected to carry out in the clinical environment.

Educators interested in replicating this intervention can adapt it to their own institutions by leveraging virtual platforms to increase accessibility, partnering with local Deaf communities and CDIs to ensure authentic representation, and embedding the session within existing curricular threads, such as Health Systems Science or clinical skills training. Based on our experiences, we encourage the use of post-session reflection through structured prompts or surveys. Further work is needed to expose healthcare trainees to Deaf culture, particularly how to work with interpreters and CDIs. Future work should explore opportunities for longitudinal integration, such as follow-up simulations or sessions with Deaf standardized patients.
